# Organizational Justice and Health Equity in Rural Japan: A Case-Based Analysis of a Hybrid Community-Hospital Organization

**DOI:** 10.7759/cureus.101921

**Published:** 2026-01-20

**Authors:** Ryuichi Ohta, Toshihiro Yakabe

**Affiliations:** 1 Community Care, Unnan City Hospital, Unnan, JPN; 2 Family Medicine, Unnan City Hospital, Unnan, JPN

**Keywords:** community–hospital collaboration, health equity, hybrid organizations, organizational justice, participatory governance, rural health

## Abstract

Rural communities face persistent health inequities driven by geographic isolation, workforce shortages, and limited access to institutional healthcare. Hybrid organizations that bridge hospitals, local governments, and communities have been proposed as promising mechanisms to address these challenges; however, empirical analyses describing how such organizations operationalize social and organizational justice remain limited. This study aimed to descriptively examine how a hybrid community-hospital organization in rural Japan operationalizes organizational justice through its structure, practices, and governance, using established frameworks of organizational design and social justice. We conducted a descriptive, theory-informed organizational case study of an anonymized non-profit organization operating in rural western Japan. This analysis did not evaluate health or service outcomes. Data sources included organizational documents, program records, and reflective analyses of ongoing activities, such as digital health consultations and community health dialogues. The analysis was guided by Mintzberg’s organizational configuration framework and Fraser’s multidimensional theory of social justice, encompassing redistribution, recognition, and representation. The organization exhibited a hybrid configuration combining elements of professional bureaucracy and adhocracy. Key activities - social networking services (SNS)-based health consultations, community health salons, and interprofessional communities of practice - illustrated how organizational structures and routines facilitated access to health information, recognition of residents’ experiential knowledge, and participatory decision-making. These practices demonstrated how organizational justice was enacted internally through relational trust and participatory processes, while externally aligning with social justice principles. However, reliance on informal coordination and volunteer labor revealed structural tensions related to sustainability and workload. This case study illustrates mechanisms through which organizational justice can be embedded in hybrid community-hospital organizations and how such designs may support health equity in rural settings. Although situated in Japan, the case offers analytically transferable insights for other rural and resource-constrained healthcare systems facing similar challenges of professional dominance, community marginalization, and institutional fragmentation. The findings highlight both the potential and limitations of justice-oriented organizational designs rather than their effectiveness in improving outcomes.

## Introduction

Rural communities continue to experience substantial health inequities arising from geographic isolation, workforce shortages, and limited access to institutional healthcare services. Addressing these complex challenges requires not only clinical interventions but also organizational arrangements that coordinate professional expertise and community participation. From an organizational design perspective, Mintzberg’s framework highlights how different configurations distribute authority, expertise, and coordination within organizations, providing a valuable lens for analyzing hybrid and community-based healthcare initiatives [1].

Within this context, community-based organizations that bridge hospitals, local governments, and residents have increasingly attracted attention as potential mechanisms for advancing health equity. Such organizations are commonly situated within the social economy, where social value creation and participatory governance are prioritized over profit generation [2,3]. The Unnan Community Health and Development Lab, located in rural western Japan, represents one such initiative. Established as a non-profit organization, the Lab functions as a hybrid entity that integrates professional healthcare expertise, participatory governance, and grassroots innovation.

Hybrid organizations must continuously negotiate multiple institutional logics, balancing professional accountability with community responsiveness and collaboration with public institutions [2,3]. In practice, these dynamics often result in organizational forms that combine elements of professional bureaucracy and adhocracy, enabling both clinical legitimacy and adaptive, project-based collaboration [1,4,5].

This study examines how the goals, organizational structure, and core activities of the Unnan Community Health and Development Lab align with its social-justice-oriented mission. To analyze this alignment, the study draws on social-justice theory, particularly Fraser’s multidimensional framework, which conceptualizes justice as encompassing redistribution, recognition, and representation [6,7]. In rural healthcare contexts, where residents’ experiential knowledge is frequently marginalized, these dimensions are especially relevant.

Innovation under resource constraints further characterizes many rural health initiatives. The distinction between bricolage and ingenieuring underscores how organizations operating in limited-resource environments rely on the creative recombination of existing social, digital, and relational resources rather than formal, resource-intensive system design [2,7,8]. While bricolage-oriented approaches may enhance adaptability and community ownership, they also raise important questions regarding sustainability and organizational accountability [9,10].

Accordingly, this study conducts a case-based organizational analysis of the Unnan Community Health and Development Lab to examine how its hybrid configuration operationalizes organizational justice and contributes to health equity in a rural setting, while also identifying structural challenges related to sustainability and governance.

## Technical report

Study design

This study employed a descriptive, case-based organizational analysis. We examined how a hybrid community-hospital organization operationalizes organizational justice and promotes health equity in a rural healthcare setting. A case study design was selected because it enables an in-depth examination of organizational structures, practices, and contextual factors in a real-world setting, particularly when organizational processes and social dynamics are central to the research question.

Study setting

This study was conducted at the Unnan Community Health and Development Lab, a legally registered non-profit organization based in Unnan City, Shimane Prefecture, a rural municipality in western Japan [11]. The Lab operates in a context characterized by physician shortages, population aging, and geographic dispersion of residents, which collectively pose challenges to timely access to healthcare and appropriate help-seeking behaviors.

The Lab was established as a community-based intermediary linking Unnan City Hospital, local government, healthcare professionals, and community residents. Its activities are designed to reach the entire population of Unnan City, with a focus on improving healthcare utilization, strengthening trust between residents and medical institutions, and addressing unmet health and medical needs through participatory approaches. The organization maintains a formal governance structure comprising nine board directors and one auditor, including a chairperson, vice-chairperson, and secretary-general, as set out in its articles of incorporation.

One of the Lab’s earliest initiatives was the development of a healthcare navigation booklet, first produced in 2013, to improve residents’ understanding of when and how to seek medical care. The booklet covered topics such as the role of primary care physicians and pharmacies, preparation for initial and follow-up visits, emergency consultation services, and residents’ roles in sustaining local healthcare systems. This booklet was distributed free of charge to all households in Unnan City and has since been used repeatedly in community meetings and educational activities.

During the COVID-19 pandemic, the Lab initiated a digital health consultation program using an open chat function on a widely used social networking platform (LINE, LY Corporation, Tokyo, Japan). This program enabled residents of all ages to seek advice from general physicians affiliated with the municipal hospital regarding personal or family health concerns. In addition to individualized responses, physicians provided daily health information posts to support health literacy. While the platform initially faced conflict and resistance among participants, continued facilitation led to the emergence of peer support and shared learning on health concerns and healthcare utilization.

In parallel, the Lab has conducted face-to-face community health dialogue sessions, referred to as “clinic salons,” since March 2022. These sessions have been held regularly in three rural districts within Unnan City - Kakeya-Tane, Kakeya-Matsukasa, and Yoshida-Fukano - and facilitated by physicians affiliated with the Lab. Dialogue themes have included at least six major health topics, such as home medical care, COVID-19 vaccination, skin diseases, physical activity and rehabilitation, appropriate use of health screenings, and cognitive decline. These sessions aimed to identify local health concerns, enhance residents’ understanding of healthcare systems, and promote trust-based relationships between residents and healthcare professionals.

Beyond direct service delivery, the Lab functions as a platform for interprofessional collaboration and participatory learning. Physicians, nurses, care managers, community members, municipal staff, and social welfare representatives meet regularly to discuss emerging health issues and outreach strategies. These activities have informed the planning of a comprehensive “medical and health consultation center,” designed to provide outreach-based responses to medical and social needs not adequately addressed by existing healthcare systems.

Operationally, the Lab conducts its activities with a total planned budget of approximately 1.63 million Japanese yen, allocated to educational materials, travel for outreach and site visits, digital infrastructure, meeting costs, and academic dissemination. This resource-constrained environment provides an empirical setting for examining how hybrid organizations employ bricolage-oriented strategies to sustain community-based healthcare initiatives.

Together, these organizational characteristics and activities situate the Unnan Community Health and Development Lab as a well-defined empirical setting in which digital consultation, community dialogue, interprofessional collaboration, and participatory governance are integrated to address rural health inequities.

Data sources

To comprehensively capture the organizational characteristics, operational practices, and justice-oriented activities of the Unnan Community Health and Development Lab, this study drew on multiple qualitative and descriptive data sources generated through the organization’s routine activities. These sources reflected both formal organizational structures and everyday practices and were selected to enable triangulation across strategic intent, program implementation, and reflective learning processes.

First, organizational documents were reviewed to clarify the Lab’s stated mission, governance structure, and strategic orientation. These documents included publicly available mission statements, internal planning documents, and written descriptions of core programs. Together, they provided foundational information on the Lab’s objectives, organizational roles, and intended mechanisms for promoting community health and equity.

Second, program records were examined to describe the scope and operational features of the Lab’s activities. These records consisted of aggregated activity data derived from routine program monitoring, including participation counts, activity frequencies, and basic descriptive summaries of digital health consultations and community-based health dialogue sessions. These data were used to characterize the scale, continuity, and modes of engagement of the Lab’s initiatives rather than to evaluate individual-level outcomes.

Third, reflective materials were analyzed to capture ongoing organizational learning and interprofessional collaboration. These materials included written summaries and notes from regular interprofessional meetings and community-of-practice discussions in which healthcare professionals, coordinators, and collaborators reflected on program implementation, ethical considerations, and emerging community needs. These reflective records provided insight into decision-making processes, adaptive responses to challenges, and the informal mechanisms through which organizational justice and participatory governance were enacted.

Collectively, these data sources enabled triangulation of organizational goals, structures, and day-to-day practices, allowing for a descriptive and theory-informed analysis of how the Lab’s hybrid organizational configuration operated in practice.

Analytical framework

Data were analyzed using a theory-informed, deductive analytical approach, in which established organizational and social-justice theories guided the interpretation of organizational structures, practices, and processes. This approach was selected to enable systematic examination of how abstract theoretical constructs were operationalized within a real-world managerial context.

First, organizational structure and coordination mechanisms were analyzed using Mintzberg’s framework for organizational configurations [5]. Organizational components (strategic apex, middle line, operating core, technostructure, and support staff) and dominant coordination mechanisms were identified from organizational documents and reflective materials. Particular attention was paid to the coexistence and interaction of professional bureaucracy, characterized by reliance on standardized professional expertise, and adhocracy, characterized by flexible, project-based collaboration. This analysis focused on how these configurations shaped decision-making processes, role distribution, and adaptive capacity.

Second, the organization’s hybrid positioning within the social economy was examined using conceptual frameworks proposed by Pearce and Kay [3,6]. The analysis explored how the Lab negotiated multiple institutional logics-public service, professional accountability, and civic participation-and how these dynamics influenced governance arrangements, resource mobilization, and relationships with external stakeholders, including hospitals and local government.

Third, justice-oriented dimensions were assessed using Fraser’s multidimensional theory of social justice, which conceptualizes justice in terms of redistribution, recognition, and representation [7]. Organizational practices and programs were deductively mapped onto these three dimensions. Redistribution was examined through mechanisms that enhanced access to health-related resources and information; recognition through practices that validated residents’ experiential knowledge; and representation through participatory decision-making and co-design processes.

Fourth, the analysis incorporated the distinction between bricolage and ingenieuring to examine innovation under resource constraints [2]. Organizational practices were assessed in terms of whether they relied on planned, resource-intensive system design or on the creative recombination of existing social, digital, and relational resources. This perspective was used to evaluate how innovation strategies influenced organizational adaptability, sustainability, and accountability.

Finally, to integrate these analytical perspectives and to examine dynamic relationships over time, a causal loop diagram (CLD) approach was applied as a supplementary systems-analysis process [12]. Key organizational variables identified through the deductive analysis-such as trust, interprofessional collaboration, organizational learning, workload, and resource constraints-were mapped into reinforcing and balancing feedback structures. The CLD was used to describe how organizational structure, justice-oriented practices, and innovation strategies interacted dynamically, highlighting feedback mechanisms related to learning and sustainability.

Analytical findings from these theoretical lenses and the causal-loop mapping were synthesized narratively, linking organizational structures and practices with justice-oriented mechanisms and observed challenges [13]. Rather than generating formal categories or quantitative measures, the analysis aimed to produce an integrative description of how organizational design, justice principles, and innovation strategies interacted within the Lab’s everyday operations.

Ethical considerations

This study analyzed organizational-level information and aggregated program data and did not involve individual-level patient data or human subject interviews conducted specifically for research purposes. All descriptions were limited to publicly available or internally approved materials. The study was conducted in accordance with ethical principles for organizational and health systems research, and no formal institutional review board approval was required.

Results

Organizational Configuration Based on Mintzberg’s Framework

Analysis of organizational documents and reflective materials indicated that the Unnan Community Health and Development Lab exhibited a hybrid organizational configuration, combining features of professional bureaucracy and adhocracy.

Elements of professional bureaucracy were evident in the reliance on certified healthcare professionals, including physicians and nurses, whose clinical expertise provided legitimacy and safety for health-related activities. Professional autonomy was maintained, particularly in clinical decision-making and ethical oversight of digital consultations and community dialogues (Table 1).

**Table 1:** Key Components of the Lab Based on Mintzberg’s Framework This table summarizes the organizational components of the Unnan Community Health and Development Lab according to Mintzberg’s framework. It outlines the roles and functions of the strategic apex, middle line, operating core, technostructure, and support staff within the Lab. The table illustrates how elements of professional bureaucracy and adhocracy coexist in this hybrid community-hospital organization, enabling both professional accountability and flexible, project-based collaboration in a resource-constrained rural setting.

Key Components	Description
Strategic apex	Hospital leadership and NPO directors (clinicians and coordinators) who set strategy and negotiate with funders
Middle line	Project managers and coordinators linking hospital, NPO, and municipal offices
Operating core	Healthcare staff, students, volunteers, and residents delivering health projects
Technostructure	Minimal bureaucracy: standardization occurs mainly through EMR integration and grant reporting
Support staff	Administrative assistants and local volunteers managing outreach and logistics

At the same time, the Lab demonstrated characteristics of an adhocracy through its flexible, project-based coordination. Activities such as digital health consultations, community health dialogues, and participatory learning initiatives were organized with minimal formal hierarchy and adapted responsively to emerging community needs. Decision-making processes were predominantly horizontal, facilitated through regular interprofessional meetings rather than formal managerial chains. The technostructure was minimal, with limited reliance on standardized procedures beyond basic documentation and coordination, enabling rapid program modification.

**Figure 1:**
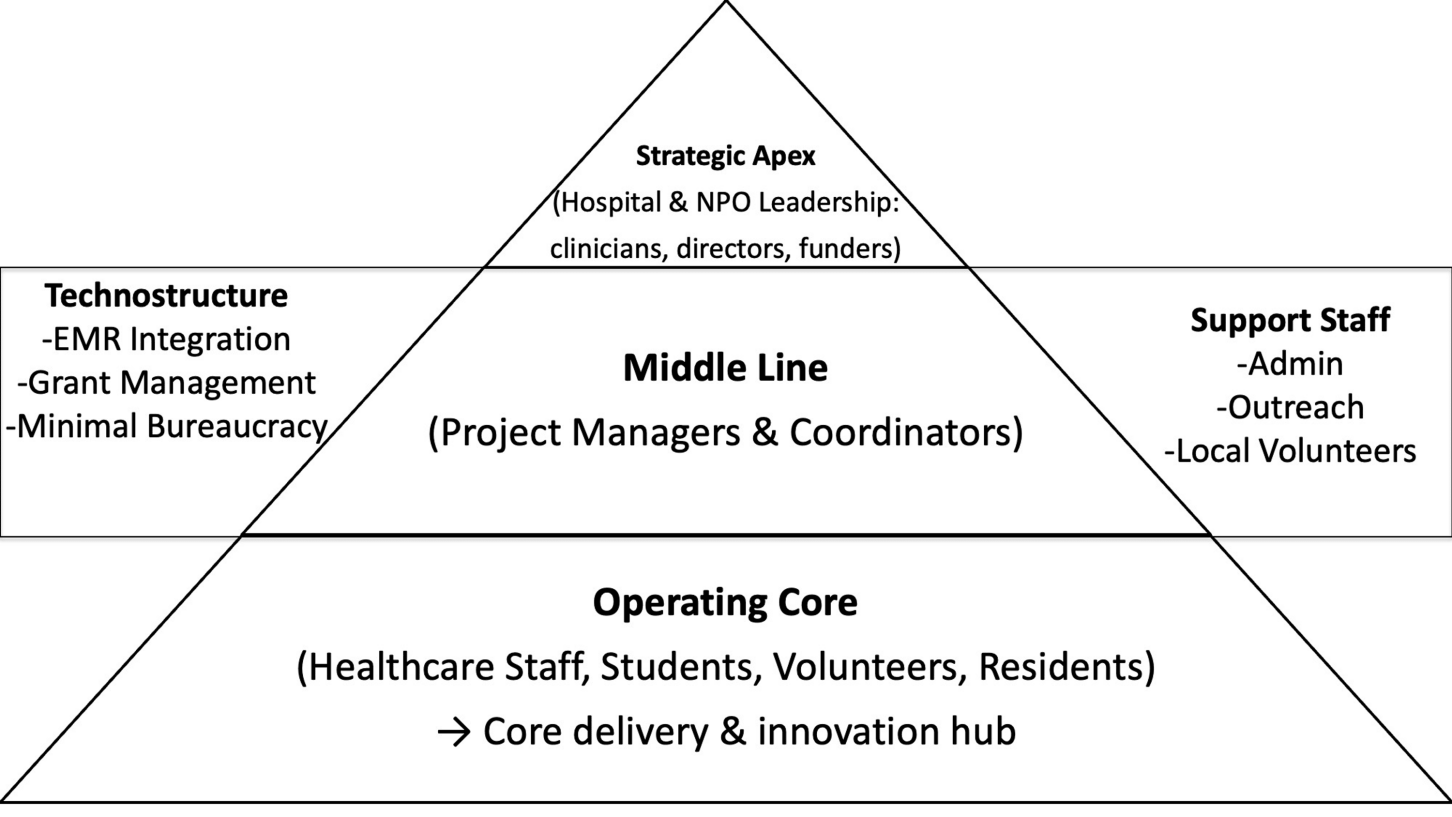
Organizational Configuration of the Unnan Community Health and Development Lab (Hybrid Model) This figure illustrates the hybrid organizational configuration of the Unnan Community Health and Development Lab based on Mintzberg’s organizational framework. The Lab integrates elements of professional bureaucracy - grounded in certified healthcare professionals and clinical accountability - with features of adhocracy characterized by flexible, project-based collaboration and horizontal decision-making. The figure highlights how strategic leadership, coordination roles, and frontline activities are interconnected to enable adaptive responses to community needs while maintaining professional legitimacy in a resource-constrained rural healthcare context. Credit, Ryuichi Ohta.

This coexistence of professional bureaucracy and adhocracy enabled the Lab to balance professional accountability with adaptive collaboration, shaping how organizational roles, responsibilities, and coordination mechanisms functioned in practice (Figure 1).

Justice-Oriented Organizational Practices Based on Fraser’s Framework

Applying Fraser’s multidimensional theory of social justice revealed that the Lab’s activities operationalized redistribution, recognition, and representation through distinct but interrelated mechanisms (Table 2).

**Table 2:** Key Components of Organizational Justice in the Lab This table summarizes key justice-oriented organizational practices of the Unnan Community Health and Development Lab using Fraser’s multidimensional framework of social justice. Organizational activities and processes are mapped onto the three interrelated dimensions of justice - redistribution, recognition, and representation - to illustrate how organizational justice is operationalized in everyday practice. The table highlights how these practices function both internally, through equitable processes and relational trust, and externally, by advancing health equity and participatory engagement in a rural community setting.

Justice	Contents
Distributive justice	Equitable sharing of workload and recognition among staff and volunteers
Procedural justice	Participatory decision-making through regular dialogue meetings
Interactional justice	Respect, transparency, and mutual trust across professional hierarchies

Redistribution was observed through initiatives that expanded access to health-related resources and information. Digital health consultations reduced geographic and logistical barriers by allowing residents to seek medical advice without visiting healthcare facilities. Community health dialogues further redistributed knowledge by providing accessible health education in familiar local settings.

Recognition was evident in practices that validated residents’ experiential knowledge. During digital consultations and community dialogues, residents’ concerns, narratives, and coping strategies were treated as legitimate inputs rather than passive recipients of professional instruction. Reflective materials documented instances in which community feedback directly informed subsequent program content, indicating reciprocal knowledge exchange between professionals and residents.

Representation was enacted through participatory governance mechanisms. Residents and non-medical participants were involved in selecting discussion themes, shaping program formats, and contributing to reflective discussions. While ultimate responsibility remained with healthcare professionals, these participatory processes created opportunities for rural residents to influence decisions affecting their health-related activities.

Innovation Under Resource Constraints: Bricolage Versus Ingenieuring

Analysis of program records and reflective materials indicated that the Lab primarily relied on bricolage-oriented innovation rather than ingenieuring. Instead of developing resource-intensive systems, the Lab repurposed existing tools and relationships, such as widely used digital communication platforms, community spaces, and voluntary professional contributions.

Digital health consultations were implemented using familiar messaging applications rather than proprietary telemedicine infrastructure. Community health dialogues were held in existing local venues, including community centers, without dedicated funding or formal facilities. Program materials were iteratively adapted based on feedback from routine activities rather than from externally designed evaluation frameworks.

These bricolage practices supported rapid implementation and adaptation of activities but also revealed organizational constraints. Reflective materials frequently noted increased workload among core members and reliance on voluntary labor, highlighting tensions between flexibility and sustainability. Accountability mechanisms remained largely relational and informal, embedded within professional norms and ongoing dialogue rather than formal reporting structures.

Causal Loop Analysis of Organizational Learning and Sustainability

**Figure 2:**
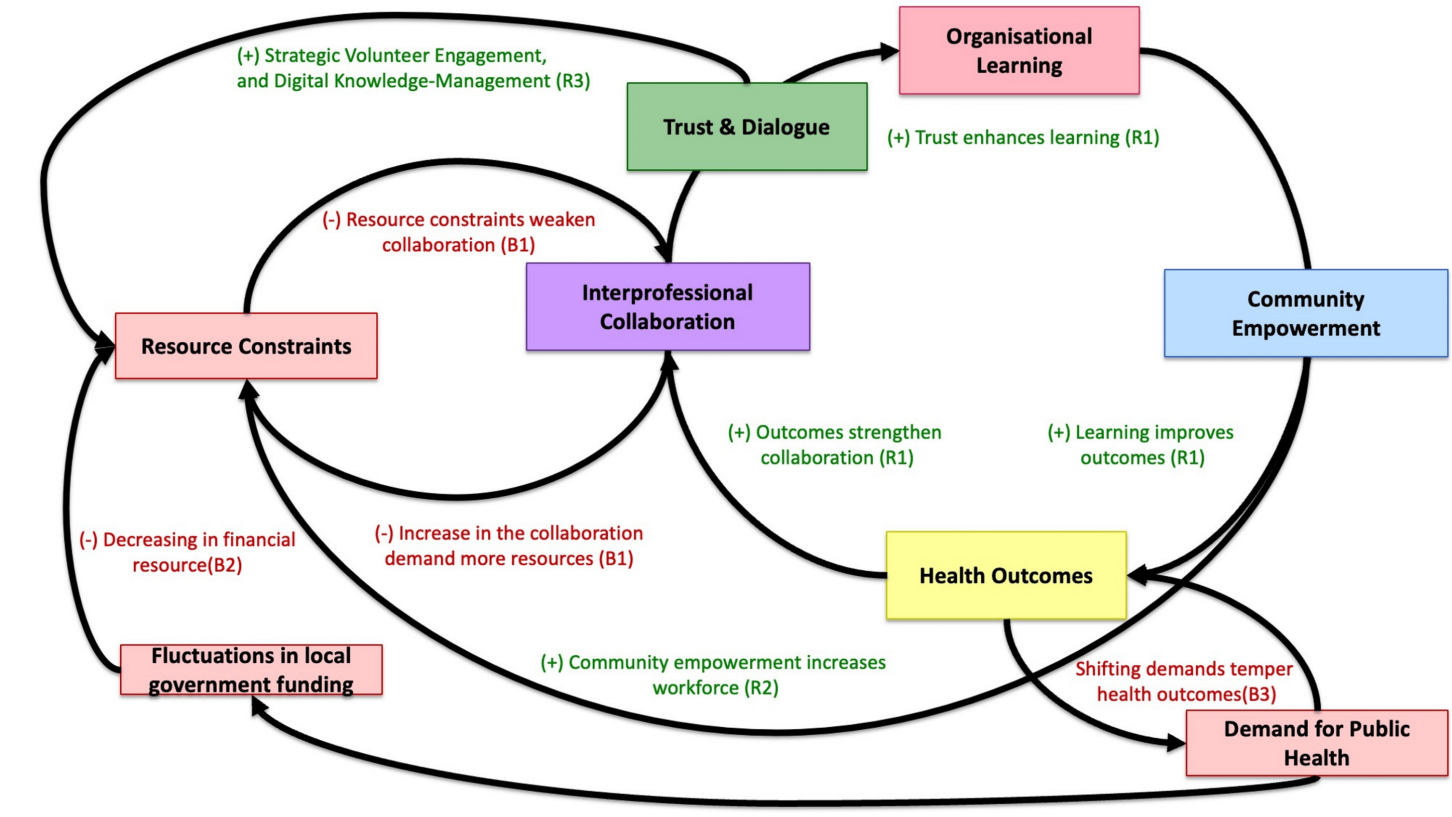
Causal Loop Diagram of Organizational Learning and Sustainability in the Lab This causal loop diagram illustrates the dynamic feedback structures shaping the Lab’s organizational learning and sustainability. The reinforcing loop (R1) depicts how trust and dialogue enhance interprofessional collaboration, strengthening organizational learning and community engagement. The balancing loop (B1) shows how increased collaboration contributes to staff workload and resource strain, moderating the reinforcing process. External balancing factors (B2, B3), including fluctuations in local government funding and shifts in public health priorities, influence system stability. Secondary reinforcing loops (R2, R3) represent resource-related and knowledge-management mechanisms that interact with the primary loops. Credit, Ryuichi Ohta.

A causal-loop mapping analysis was conducted to describe the dynamic interactions among the Lab’s organizational activities, learning processes, and sustainability (Figure 2).

## Discussion

This case-based organizational analysis illustrates how a hybrid community-hospital organization can operationalize organizational justice and contribute to health equity in a rural healthcare setting. By integrating professional expertise, participatory governance, and bricolage-oriented innovation, the Unnan Community Health and Development Lab demonstrates how justice-oriented organizational principles can be embedded within everyday practice rather than implemented solely through formal policy or institutional reform.

From an organizational design perspective, the Lab’s hybrid configuration - combining elements of professional bureaucracy and adhocracy - appears central to its adaptive capacity. Professional bureaucracy provided clinical legitimacy, ethical oversight, and safety, while adhocratic features enabled flexible coordination and rapid responsiveness to community needs [14,15]. Rather than resolving tensions between accountability and flexibility, the Lab sustained a fluid organizational form in which professional standards coexisted with horizontal collaboration. This finding supports theoretical perspectives that view hybridity not as a transitional state but as a durable organizational response to complex social and healthcare challenges in resource-constrained rural contexts [16].

Applying Fraser’s multidimensional framework clarifies how social justice was operationalized through organizational practices. Redistribution was enacted through mechanisms that lowered barriers to accessing health-related information and support, particularly for geographically or socially isolated residents [17]. Recognition was embedded in practices that treated residents’ experiential knowledge as legitimate and actionable, countering hierarchical knowledge relations common in institutional healthcare [18]. Representation was facilitated through participatory decision-making and co-design processes, enabling residents to influence program content and priorities [19]. Notably, these justice-oriented practices emerged from routine organizational processes rather than from discrete interventions, suggesting that organizational justice may be a prerequisite for broader social justice outcomes in rural healthcare.

The analysis also highlights the central role of bricolage in shaping innovation under resource constraints. By repurposing existing digital platforms, community spaces, and professional goodwill, the Lab implemented and sustained multiple initiatives without substantial financial investment. This bricolage-oriented approach enhanced adaptability, reduced barriers to participation, and strengthened community ownership [20]. However, the causal-loop analysis revealed that structural tensions accompanied these advantages [12]. The reinforcing loop linking trust, collaboration, and organizational learning supported continued engagement while simultaneously activating balancing dynamics related to workload, fatigue, and limited resources.

The causal-loop perspective adds a dynamic dimension to understanding sustainability. Rather than treating success and strain as separate phenomena, the feedback structure illustrates their intrinsic connection [13]. Growth in trust-driven collaboration both enables learning and increases organizational burden, indicating that sustainability is not a static attribute but a continuously negotiated balance between expansion and protection against overload [11]. External factors, including fluctuations in local government funding and shifting public health priorities, further modulate these dynamics, underscoring that community-based organizations operate within broader policy ecosystems [11].

Together, these findings suggest that advancing health equity in rural settings requires attention not only to service delivery models but also to organizational design and justice. Hybrid community-hospital organizations may serve as effective intermediaries when they foster participatory governance and relational trust. However, sustaining such models requires deliberate attention to governance structures, workload distribution, and partnership mechanisms that buffer against resource strain [10]. Without such support, the very processes that promote equity may contribute to organizational fragility.

This study has several limitations. As a single-case analysis, the findings are not statistically generalizable. The analysis relied on organizational documents, program records, and reflective materials rather than prospective data collection or stakeholder interviews explicitly conducted for research purposes. In addition, the causal-loop diagram serves as a heuristic tool rather than quantifying causal effects. Nevertheless, the study offers analytical generalizability by illustrating mechanisms through which organizational justice and health equity may be linked in rural healthcare contexts.

## Conclusions

This study demonstrates that organizational justice can function as a foundational mechanism for advancing health equity within hybrid community-hospital organizations. By integrating professional expertise, participatory governance, and bricolage-driven innovation, the Unnan Community Health and Development Lab illustrates how justice-oriented organizational design can be enacted in everyday practice, while also highlighting the importance of actively managing sustainability and organizational strain in rural healthcare systems.
